# Exposure to Bisphenol A Caused Hepatoxicity and Intestinal Flora Disorder in Rats

**DOI:** 10.3390/ijms23148042

**Published:** 2022-07-21

**Authors:** Ruijing Liu, Boping Liu, Lingmin Tian, Xinwei Jiang, Xusheng Li, Dongbao Cai, Jianxia Sun, Weibin Bai, Yulong Jin

**Affiliations:** 1 Key Laboratory for Bio-Based Materials and Energy of Ministry of Education, College of Materials and Energy, South China Agricultural University, Guangzhou 510630, China; liuruijing1000@126.com (R.L.); boping@scau.edu.cn (B.L.); 2 Department of Food Science and Engineering, Institute of Food Safety and Nutrition, Guangdong Engineering Technology Center of Food Safety Molecular Rapid Detection, Jinan University, Guangzhou 510632, China; tianlinmin@163.com (L.T.); haiyuanjxw@126.com (X.J.); lixusheng1016@163.com (X.L.); caidongbao1123@163.com (D.C.); 3 School of Chemical Engineering and Light Industry, Guangdong University of Technology, Guangzhou 510006, China; jxsun1220@163.com

**Keywords:** bisphenol A, hepatoxicity, SIRT/PGC-1α, apoptosis, gut microbiota, SCFAs

## Abstract

Bisphenol A (BPA) is a globally utilized industrial chemical and is commonly used as a monomer of polycarbonate plastics and epoxy resins. Recent research reveals that BPA could cause potential adverse biological effects and liver dysfunction. However, the underlying mechanisms of BPA-induced hepatoxicity and gut dysbiosis remain unclear and deserve further study. In this study, male Sprague Dawley rats were exposed to different doses (0, 30, 90, and 270 mg/kg bw) of BPA by gavage for 30 days. The results showed that the high dose of BPA decreased superoxide dismutase (SOD), glutathione (GSH), and increased malondialdehyde (MDA) levels. Moreover, a high dose of BPA caused a significant increase in serum alanine aminotransferase (ALT), aspartate aminotransferase (AST), total cholesterol (TC), and low-density lipoprotein cholesterol (LDL-C), while high-density lipoprotein cholesterol (HDL-C) was significantly decreased in BPA-treated rats. The gene expression of PGC-1α and Nrf1 were decreased in the liver of high doses of BPA-administrated rats, as well as the protein levels of SIRT1, PGC-1α, Nrf2, and TFAM. However, the protein expression of IL-1β was significantly increased in BPA-treated rats. In addition, BPA weakened the mitochondrial function of hepatocytes and promoted cell apoptosis in the liver by up-regulating the protein levels of Bax, cleaved-Caspase3, and cleaved-PARP1 while down-regulating the Bcl-2 in the liver. More importantly, a high dose of BPA caused a dramatic change in microbiota structure, as characterized at the genus level by increasing the ratio of *Firmicutes* to *Bacteroidetes* (F/B), and the relative abundance of *Proteobacteria* in feces, while decreasing the relative abundance of *Prevotella_9* and *Ruminococcaceae_UCG-014,* which is positively correlated with the content of short-chain fatty acids (SCFAs). In summary, our data indicated that BPA exposure caused hepatoxicity through apoptosis and the SIRT1/PGC-1α pathway. BPA-induced intestinal flora and SCFA changes may be associated with hepatic damage. The results of this study provide a new sight for the understanding of BPA-induced hepatoxicity.

## 1. Introduction

Bisphenol A (BPA) is a high-volume organic synthetic compound monomer widely used in the synthesis of epoxy resin and polycarbonate plastics, such as food and beverage packaging, medical devices, thermal paper, and dental materials [1]. Therefore, humans are exposed to BPA in a variety of ways, including in drinking water, food, air, and other ways. After entering the human body, BPA can accumulate in human tissues and is potentially harmful to human health through different molecular mechanisms [2]. Studies have shown that BPA can be detected in blood, urine, amniotic fluid, placenta, cord blood, and human breast milk at different concentrations; thus, BPA exposure is considered an inevitable situation, and it may cause liver dysfunction, chronic diseases, obesity, cancer, reproductive toxicity, and diabetes [3,4,5,6,7].

The liver is the largest and most metabolically complex organ in the body. It is a vital organ responsible for the detoxification and metabolism of xenobiotics in humans [8]. The process of liver metabolism of exogenous poisons will lead to the production of free radicals, which are very active and unstable. When the production of free radicals exceeds the range of the body’s scavenging capacity, the imbalance between the production and elimination of free radicals will lead to oxidative stress and eventually cause liver injury [9]. In addition, the liver is the main organ that is responsible for BPA metabolism and transforms BPA into glucuronidation form in animals and humans. Therefore, it is more susceptible to BPA than other organs [10]. Sirtuins are evolutionarily conserved NAD+-dependent class III histone deacetylase. It is one of the silent-information-regulator 2 (Sir2) superfamily and plays important role in a broad range of biological activities [11]. As reported, seven sirtuin isoforms, SIRT1–7, have been identified in humans [12]. In recent years, Sirtuin 1 (SIRT1) is the most widely studied sirtuin protein, and it is also a popular drug design target. Furthermore, SIRT1 plays a vital role in many physiological functions such as energy metabolism, inflammation, oxidative stress response, neuronal signaling, cell survival, mitochondrial biogenesis, and apoptosis [13,14]. SIRT1 can also regulate hepatic metabolism by deacetylating key metabolic factors such as peroxisome proliferator-activated receptor-gamma (PPARγ)-coactivator 1alpha (PGC-1α) [15]. As a deacetylation substrate of SIRT1, PGC-1α can regulate nuclear and mitochondria transcription factors, including nuclear respiratory factor (Nrf1), nuclear factor E2-related factor 2 (Nrf2), and mitochondrial transcription factor A (TFAM) [16]. Nrf1 activates the expression of key factors in regulating cellular respiration, mitochondrial DNA replication, and transcription, while TFAM directly binds to mitochondrial DNA and is required for the maintenance of mitochondrial DNA [17]. Nrf2 is the master regulator of the antioxidant responsive element (ARE)-mediated induction of phase II detoxification and antioxidative enzyme gene expression [18]. Therefore, Nrf2 plays an important role in maintaining redox balance in the liver.

The gut microbiota is a complex microbial community that has a crucial impact on human physiological processes such as immune regulation, energy balance, information exchange, and gastrointestinal development [19]. Furthermore, it is an important source of metabolites, hormones, and neuro-mediators that directly regulate gut function and indirectly modulate the function of extra-intestinal organs such as the liver, brain, and kidney. When the gut barrier is damaged, its permeability increases, leading to automatic exposure to multiple harmful substances and bacteria from the gut to the liver [20]. Due to the widespread use of BPA, some investigations have shown the adverse effects of BPA in both humans and animals. A recent investigation has shown that hepatotoxicity following BPA exposure is associated with mitochondrial oxidative stress and dysfunction. Previous studies also have shown that BPA can induce the generation of reactive oxygen species (ROS) as well as functional and structural changes in the liver of mice and rats [21,22]. However, the in-depth mechanisms of BPA-induced liver injury and intestinal dysfunction remain unclear, and the signaling pathway of injury induction needs to be illustrated.

The goals of the present study were to investigate the mechanisms of BPA-induced liver toxicity and intestinal disorders in rats. The results of this study may provide novel insight into BPA-induced liver toxicity and extend our knowledge by exploring the relationships between liver injury and disturbance of intestinal flora.

## 2. Results

### 2.1. Effects of BPA on Liver Weight and Oxidative Stress

**Figure 1:**
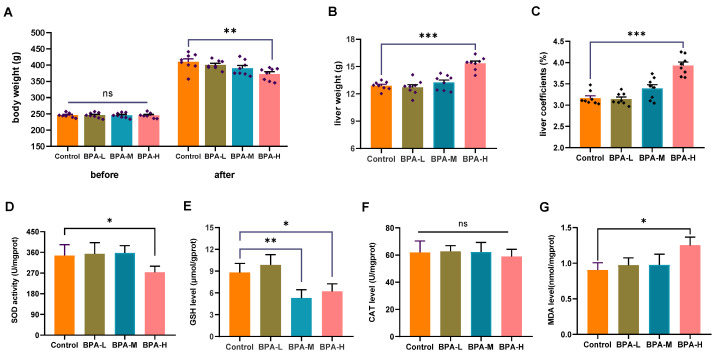
Effects of BPA on liver oxidative stress in rats. (**A**) Changes in body weight before and after the experiment. (**B**) Liver weight and (**C**) liver coefficients of rats. (**D**) SOD, (**E**) GSH, (**F**) CAT, and (**G**) MDA in BPA-treated rats. Data are shown as the mean ± SEM (*n*  =  8). * *p* < 0.05, ** *p* < 0.01, *** *p* < 0.001 compared with the Control group. ns: no significance.

As shown in Figure 1A, there was no difference in the initial body weight among the experimental groups. However, the mean body weight of rats in the high dose of BPA (BPA-H) group markedly decreased compared with the Control group at the end of the experiment (*p* < 0.01). The liver weight and coefficient of rats in the BPA-H group were higher than that in the Control group (*p* < 0.001) (Figure 1B,C). In addition, a high dosage of BPA significantly reduced SOD activity in rat liver (*p* < 0.05) (Figure 1D), as well as the level of GSH in medium dose of BPA (BPA-M) and BPA-H groups (*p* < 0.05, *p* < 0.01) (Figure 1E). No changes were observed in CAT levels (Figure 1F). A significant increase in MDA levels was observed after treatment with a high dosage of BPA (*p* < 0.05) (Figure 1G).

### 2.2. Effects of BPA on Serological Markers

**Figure 2:**
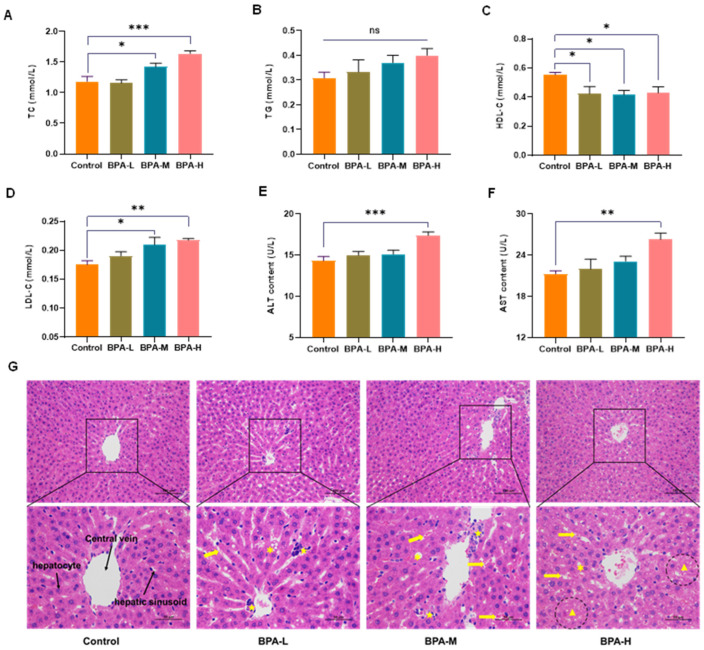
Effects of BPA on serum levels of (**A**) TC, (**B**) TG, (**C**) HDL-C, (**D**) LDL-C, (**E**) ALT, and (**F**) AST. These data were expressed as means ± SEM (*n* = 8). * *p* < 0.05, ** *p* < 0.01 and *** *p* < 0.001 versus the Control. Histological sections of the liver were stained with hematoxylin-eosin (HE) (200×, 400×) (**G**). ✱ steatosis; Yellow arrows: sinusoidal dilatation and congestion; ★ infiltration of inflammatory cells; ▲ necrosis. ns: no significance.

In the medium and high dose of BPA groups, the serum levels of TC and LDL-C increased significantly (*p* < 0.05, *p* < 0.01), while the concentration of HDL-C decreased significantly in the BPA-M group (*p* < 0.05) compared with the Control group (Figure 2A,C,D). No significant change in TG levels (Figure 2B). Clinically, AST and ALT are two critical biomarkers commonly used to reflect liver function [23]. As shown in Figure 2E,F, the levels of AST and ALT in serum were increased markedly in the BPA-H group (*p* < 0.01). According to the above results, BPA exposure can lead to liver injury.

### 2.3. BPA-Induced Liver Histopathological Damage

Next, HE staining was used to evaluate the effect of BPA on liver damage. The histopathological alterations in the liver of rats from different experimental groups are shown in Figure 2G. In the Control group, the liver presented normal structures with neatly arranged hepatocytes and regular hepatic sinusoids. However, BPA exposure damaged liver tissues, characterized by hepatic cord derangement, expansion of hepatic sinusoid between hepatic cords, congestion in sinusoidal spaces, degeneration of hepatocytes, and infiltration of inflammatory cells. Meanwhile, hepatocyte necrosis and severe congestion of hepatic sinusoid were observed in the high dose of BPA rats compared to the Control group.

### 2.4. Effects of BPA on SIRT1/PGC-1α Pathway

**Figure 3:**
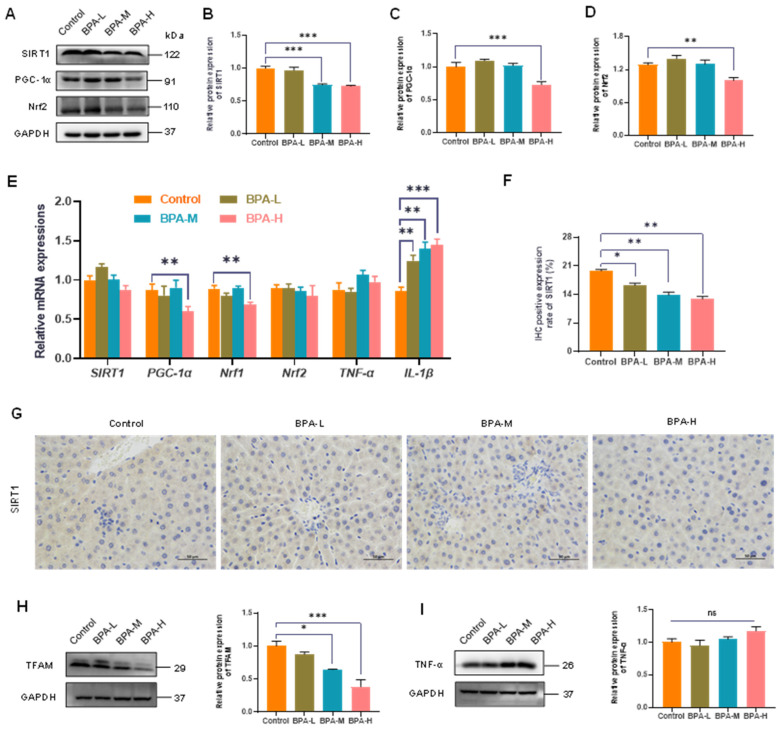
Effects of BPA on the liver SIRT1/PGC-1α pathway. (**A**) The relative protein levels of PGC-1α, Nrf2, and SIRT1. (**B**–**D**) Values of quantitative analysis (*n* = 4). (**E**) Relative mRNA levels of SIRT1, PGC-1α, Nrf1, Nrf2, TNF-α, and IL-1β. (**F**) Percentage of immunostaining for Sirt1. (**G**) Immunohistochemistry shows the expression of hepatic SIRT1 (400× magnifications). (**H**) The relative protein levels of TFAM and (**I**) the relative protein levels of TNF-α. Data are presented as mean ± SEM. * *p* < 0.05, ** *p* < 0.01, *** *p* < 0.01 vs. control group. ns: no significance.

The SIRT1-mediated PGC-1α pathway plays an important role in regulating the body’s antioxidant capacity and mitochondrial production and functional status in the toxic damage caused by some poisons [24]. Therefore, we further investigated whether the PGC-1α and SIRT1 were involved in the corresponding responses of liver injury caused by BPA exposure. The protein expressions of SIRT1 in the BPA-M and H groups, and of PGC-1α in the BPA-H group, were downregulated (*p* < 0.001) (Figure 3A–C). Meanwhile, the protein level of Nrf2, downstream of PGC-1α, was decreased in BPA-H groups (*p* < 0.01) (Figure 3D). Accordingly, the mRNA expression of PGC-1α and Nrf1 significantly reduced after a high dosage of BPA treatment (*p* < 0.01). Moreover, the mRNA expression of IL-1β in rat liver was significantly increased in BPA-treated rats (*p* < 0.01, *p* < 0.001) (Figure 3E). To further investigate the mechanism of hepatotoxicity caused by BPA, SIRT1 proteins were determined by IHC. There was a significant decrease in the expression of SIRT1 in the BPA-treated groups (*p* < 0.05, *p* < 0.01) (Figure 3F,G). Furthermore, TFAM was significantly reduced in BPA-M, H groups (*p* < 0.05) (Figure 3H), while no significant change was observed in TNF-α levels when compared to the Control group (Figure 3I). These data together suggested that the SIRT1/PGC-1α pathway was indeed involved in liver injury caused by BPA.

### 2.5. BPA-Induced Hepatocyte Apoptosis in Liver

**Figure 4:**
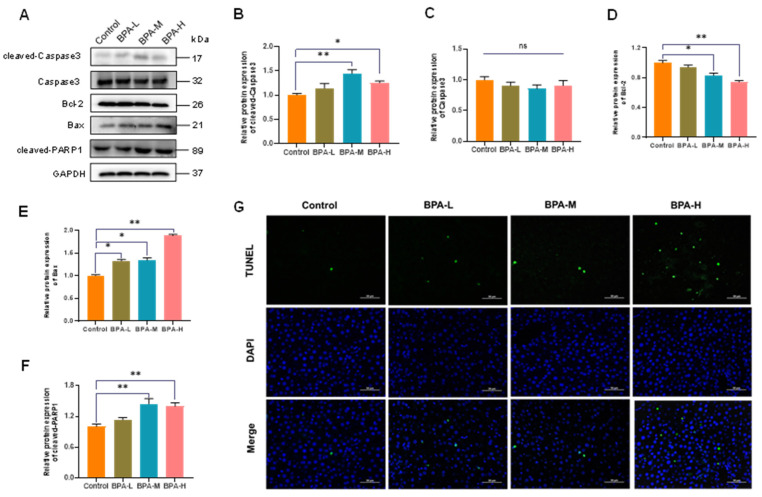
Effects of BPA on apoptosis in the liver from mice treated for 30 days. (**A**) Western blot analysis of apoptosis-related protein levels, and (**B**–**F**) grey-scale of quantitative analysis. Values are mean ± SEM (*n* = 4). * *p* < 0.05, ** *p* < 0.01, compared to the Control group. ns: no significance. (**G**) Representative TUNEL images of liver tissues.

Apoptosis is important to ensure liver tissue homeostasis during normal cell turnover and to control liver growth and regeneration [25]; additionally, the intrinsic pathway of apoptosis is closely regulated by the Bcl-2 family of proteins [26]. Bcl-2 and Bax are important proteins in the mitochondrial-mediated apoptotic pathway. Moreover, cleaved-Caspase3 is a classic gene involved in the terminal stages of cell apoptosis [27]. In this study, BPA exposure induced the protein expressions of cleaved-Caspase3 in the rat liver (*p* < 0.05, *p* < 0.01) (Figure 4A,B), while there was no significant change in Caspase3 (Figure 4C). The protein level of Bcl-2 in the liver was significantly decreased in BPA-M and H group (*p* < 0.05, *p* < 0.01) (Figure 4D), while the Bax protein expression was significantly increased after exposure to BPA (*p* < 0.05, *p* < 0.01) (Figure 4E). In addition, BPA exposure induced the protein expressions of cleaved-PARP1 in medium and high doses of BPA groups (*p* < 0.01) (Figure 4F). Furthermore, hepatocyte apoptosis in liver sections was subsequently detected by TUNEL staining. The results showed that the high dose of BPA exposure increased liver TUNEL-positive cells in the rats (Figure 4G).

### 2.6. Effects of BPA on α and β Diversity of Gut Microbiota in Rats

**Figure 5:**
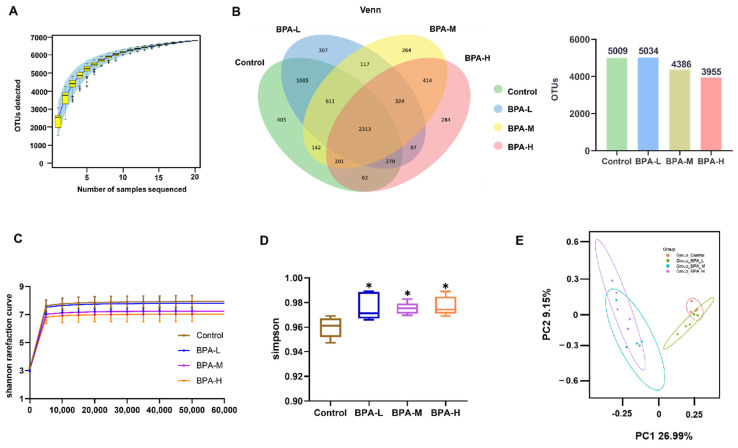
Effects of BPA on the structure of gut microbiota. (**A**) OTU numbers. (**B**) Venn diagram illustrated the unique and shared OTUs among groups. (**C**) Shannon rarefaction curve, (**D**) Simpson index, and (**E**) PCA of gut microbiota communities. * *p* < 0.05 vs. Control group.

To explore the changes in the gut microbiota after administration of BPA in rats, the abundance and composition of microbiota were analyzed by high-throughput sequencing of the V3 and V4 regions of the 16S rRNA gene in the fecal contents. The rarefaction curve of each sample was consistent with the species accumulation curves (Figure 5A). The Venn diagram displays the unique and shared OTUs among four groups. It was found that the number of OTUs in the BPA-M and H groups was less than that in the Control group (Figure 5B). With the increase of gene sequences, the Shannon rarefaction curves of all groups tended to be stable, which proved the reliability of sequencing data. Shannon rarefaction curves showed significant separation between the BPA-treated rats and the Control group (Figure 5C). Compared with the Control group, the α diversity index of richness (Simpson) was significantly increased in rats after administration of BPA (Figure 5D). In addition, β diversity was analyzed by principal component analysis (PCA) (Figure 5E). The results showed significantly separated clustering of the gut microbiota structure between the Control group and the BPA groups, suggesting that the whole microbial community structure was changed due to BPA exposure.

### 2.7. Effects of BPA on the Community of Gut Microbiota in Rats

**Figure 6:**
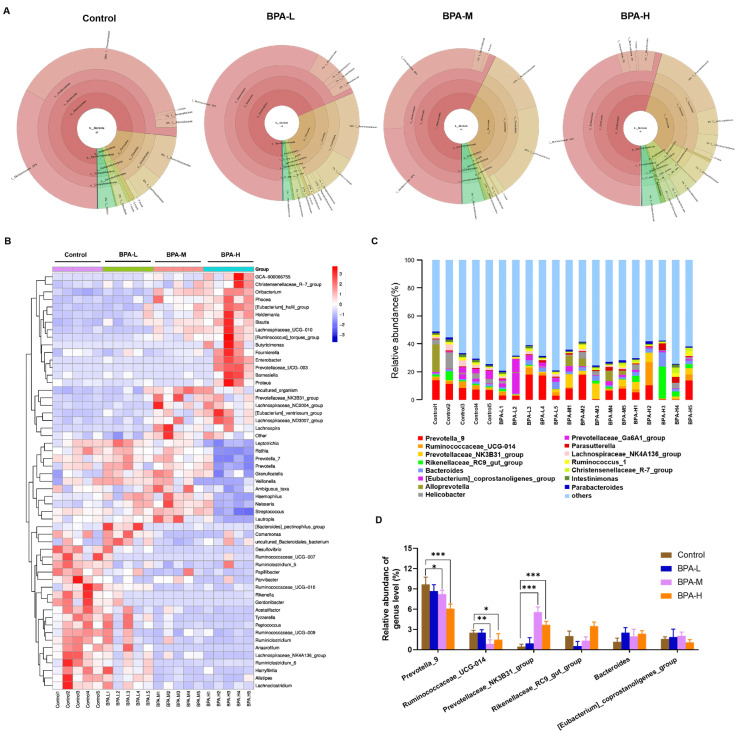
Relative abundance of microbial species at the genus level of rats’ feces. (**A**) Relative abundance of bacteria at different levels displayed using Krona. (**B**) The difference in feces bacterial structure at the genus level among different groups. (**C**) The top 15 species of each group in terms of maximum abundance on the genus level. (**D**) The higher bacterial community at the genus level in rats. Data are shown as mean ± SEM. * *p* < 0.05, ** *p* < 0.01, *** *p* < 0.001 vs. Control group.

To determine whether there were differences in the structure of the gut microbiota, we used Krona to illustrate the abundance of gut microbiota composition in each group at different classification levels. At the phylum level, Bacteroidetes (71% vs. 69% vs. 57% vs. 54%), and Firmicutes (16% vs. 26% vs. 36% vs. 33%) are the main dominant phyla followed by Proteobacteria (3% vs. 3% vs. 2% vs. 8%), and Epsilonbacteraeota (4% vs. 2% vs. 5% vs. 4%) for the Control, low dosage of BPA (BPA-L), BPA-M, and BPA-H groups, respectively. Bacteroidetes was the most abundant phylum in feces, and the relative abundance was the highest in the Control group. A high dose of BPA administration caused the relative abundance of Bacteroidetes to decrease by 17%, while Firmicutes increased by 17% (Figure 6A). According to the abundance information in each sample, hierarchical cluster analysis of the microbial profiles at the genus level indicated which species are more or less concentrated in which samples. The microbial spectrum of BPA groups was different from that of the control group. Oribacterium, Enterobacter, Prevotellaceae_UCG-003, Lachnospiraceae_UCG-010, Eubacterium_hallii_group, Phocea, Blautia, Holdemania, Butyricimonas, Barnesiella, Proteus, Ruminococcus_torques_group, and Fournierella were mainly clustered in the BPA-H group, whereas Alistipes, Anaerofilum, Desulfovibrio, Lachnoclostridium, Lachnospiraceae_NK4A136_group, Papillibacter, Parvibacter, Peptococcus, and Ruminiclostridium were gathered in the Control group (Figure 6B). Moreover, the most abundant OTUs with statistical differences at the genus level are shown in Figure 6C,D. BPA exposure significantly decreased the abundance of Prevotella_9 (*p* < 0.05, *p* < 0.001) and Ruminococcaceae_UCG-014 (*p* < 0.05, *p* < 0.01), while the abundance of Prevotellaceae_NK3B31_group was significantly increased (*p* < 0.001).

### 2.8. Effects of BPA on Fecal SCFA Levels in Rats

**Figure 7:**
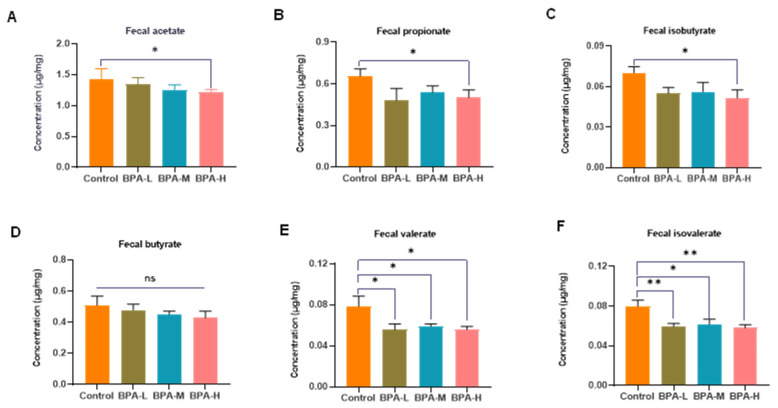
Effects of BPA on the fecal SCFA levels. (**A**) Acetate, (**B**) propionate, (**C**) isobutyrate, (**D**) butyrate, (**E**) valerate, and (**F**) isovalerate. Values were expressed as mean ± SEM in each group (*n* = 8). * *p* < 0.05, ** *p* < 0.01 compared with the Control group. ns: no significance.

As the major microbial fermentation products of diets, SCFAs can reduce the pH of the colon, inhibit pathogens, and regulate intestinal mucosal barrier function [28]. To study the possible differences in SCFA production, the concentrations of SCFAs (acetate, propionate, isobutyrate, butyrate, valerate, and isovalerate) in rat feces were analyzed by GC to display the possible effects of BPA. Compared to the control group, the concentrations of acetate (1.43 ± 0.37 vs. 1.21 ± 0.11) (Figure 7A), propionate (0.65 ± 0.12 vs. 0.50 ± 0.12) (Figure 7B), isobutyrate (0.07 ± 0.01 vs. 0.05 ± 0.01) (Figure 7C), valerate (0.08 ± 0.02 vs. 0.06 ± 0.01) (Figure 7E), and isovalerate (0.08 ± 0.01 vs. 0.06 ± 0.01) (Figure 7F) were markedly decreased after treatment with high-dose BPA (*p* < 0.05, *p* < 0.01). No significant change in butyrate levels was observed (Figure 7D).

## 3. Discussion

BPA is considered an endocrine disruptor and concerns are raised by the general public over its endocrine-disruptive effects of it [29]. Thus, BPA exposure induces a spectrum of toxic effects including cancer, infertility, diabetes, and obesity. The liver is the main organ for catabolizing exogenous compounds, which also means it is particularly susceptible to injury from xenobiotics [30]. Considering that the underlying mechanism of BPA-induced hepatoxicity is still unclear, and its relationship with the gut microbiota is not clear, we carried out the present study to investigate the toxic effects of BPA on the liver and the composition of intestinal microbiota in rats. Our results suggested that BPA exposure produced hepatotoxicity and disturbed gut microbiota. The mechanism of liver injury may be through inhibiting the SIRT1/PGC-1α pathway while promoting hepatocyte apoptosis, leading to the progression of tissue damage.

Body weight can usually be used as one of the basic indicators to evaluate the growth, development, and energy metabolism of the body. Body weight may change when stimulated by exogenous toxins. In the present study, the high dose of BPA-treated groups of rats showed a decrease in body weight compared to the Control group, suggesting that the BPA had adverse effects on the rats. The result is similar to the study of Wang et al [31]. In addition, the liver coefficient is the ratio of liver weight to body weight, which is a common index in toxicology experiments. The increase in the liver weight and coefficient indirectly reflects the swelling, congestion, and hypertrophy of the liver when exposed to BPA, which is consistent with the pathological result. On the other hand, some reports were inconsistent with our findings, the variation in results may be attributed to the diversity in diet composition, BPA exposure period, route, and doses [32,33,34].

Oxidative stress is an important indicator of liver injury, which reflects an imbalance in the redox system [35]. Oxidative stress is mainly caused by an excess accumulation of free radicals, especially ROS, and a decrease in antioxidant defense, a process that initiates and promotes liver damage [36]. Excessive accumulation of ROS in the body can lead to DNA damage, lipid accumulation, and ultimately cell damage or death [37]. Antioxidants can usually reduce cellular injury resulting from the interaction between lipid, protein, and DNA molecules and ROS [38]. Therefore, antioxidant indicators such as SOD and CAT are frequently used to evaluate oxidative stress caused by environmental pollutants. Besides, as a co-substrate in the detoxification response of some xenobiotics by glutathione peroxidase (GSH-Px) and glutathione S-transferase (GST), GSH deficiency is also a major signal of oxidative stress [39]. Interestingly, in the current study, the results showed that a high dose of BPA exposure decreased SOD and GSH activities while increasing MDA levels. The results were in concordance with a previous study [38]. It is speculated that the low levels of antioxidant enzymes in the liver may be caused by scavenging the free radicals. Aminotransferases (AST and ALT) are the most widely used biomarkers in clinical trials to assess hepatotoxicity [40]. The present study found that administration of a high dose of BPA significantly increased the concentrations of serum AST and ALT, as well as the levels of TC and LDL-C Accordingly, a previous study revealed that exposure to BPA increased the hepatic TC and TG contents, and up-regulated the expression of genes associated with lipid synthesis in male C57BL/6 mice [41]. The liver enzymes ALT, AST, alkaline phosphatase (ALP), and lactate dehydrogenase (LDH) are released into the bloodstream following inflammation and necrosis when the liver is damaged. In the present study, the pathological findings showed that BPA treatment caused liver tissue dilatation of sinusoids, congestion, inflammation, and necrosis in a dose-dependent manner. Taken together, the biochemical indicators and histopathology results of our study suggest that BPA exposure induced liver damage in rats.

Apoptosis is a form of programmed cell death, which maintains the normal development of tissues and homeostasis by eliminating unnecessary or abnormal cells [42]. However, as one of the main features of acute-chronic diseases and intoxications, abnormal apoptosis can also be triggered by external factors such as environmental pollutants. Different species have different mechanisms for regulating cell death, but they are all regulated by homologous proteins and mitochondria. Moreover, mitochondria are involved in the integration and circulation of intracellular death signals, including oxidative stress and apoptosis [43]. A previous study has shown that liver apoptosis induced by BPA is associated with mitochondrial oxidative stress and dysfunction [44]. In this study, we observed higher levels of Bax, cleaved-Caspase3, and cleaved-PARP1 in BPA exposure groups. However, the protein expression of Bcl-2 in the liver was significantly reduced in BPA-M and BPA-H groups. Accordingly, our data also showed that the proportion of apoptotic cells was significantly increased following BPA treatment. The pro-apoptotic factor Bax and the anti-apoptotic factor Bcl-2 play crucial roles in regulating mitochondria-dependent apoptosis. Bcl-2 exerts anti-apoptotic effects by inhibiting the pro-apoptotic protein produced by Bax that can penetrate the outer membrane of the mitochondria, thereby inhibiting the release of cytochrome C to the cytoplasm and the cascade [45,46]. Therefore, our data thus suggested that BPA can induce apoptosis of the liver via the mitochondria pathway.

PGC-1α acts as a transcriptional co-activator, coordinating the activities of various transcription factors involved in mitochondrial proliferation; therefore, it is accepted as a central regulator [47]. SIRT1, the mammalian ortholog of yeast Sir 2, is mainly concentrated in the nucleus and can regulate the function of PGC-1α by regulating the acetylation of PGC-1α. In addition, the downstream transcription factors including PPAR, Nrf, and TFAM of PGC-1α can also be regulated, further influencing mitochondrial biogenesis and function [48]. Mitochondrial biogenesis can help mitigate deleterious consequences of oxidative stress. Therefore, we analyzed the gene and protein expressions of SIRT1, PGC-1α, Nrf1, Nrf2, and TFAM in the liver. We found that BPA significantly down-regulated the protein levels of SIRT1, PGC-1α, Nrf2, and TFAM, as well as the gene expressions of PGC-1α and Nrf1. The results suggested that inhibition of the SIRT1/PGC-1α pathway will impair the quality of mitochondrial synthesis, thereby exacerbating oxidative stress. Similarly, a previous study reported that BPA exposure led to the decrease of PGC-1α and TFAM expressions, and impaired mitochondrial biogenesis, therefore resulting in neurotoxicity [49]. In addition, previous research has also reported that the level of SIRT1 gene expression was significantly decreased in the rat after BPA exposure than that in the control groups [50]. Taken together, our results together with others suggested inhibiting the SIRT1-mediated PGC-1α pathway can reduce the antioxidant capacity of tissues and impair mitochondrial production and functional status. 

Gut microbiota is involved in various physiological activities of organisms, including metabolism, absorption, and production. The main metabolites produced by the gut microbiota are SCFAs, which are the end products of dietary fiber fermentation in the gut. The levels of SCFAs in the intestine are greatly influenced by the intestinal flora, and the imbalance of intestinal flora can lead to the disproportion of SCFAs produced. Acetic acid, propionic acid, and butyric acid make up 95% of SCFAs in the human gut [51]. The interaction between the gut and liver is mediated through the portal vein. It can transfer the gut-derived products including nutrients, microbial metabolites, and microbial components to the liver. Then these components enter the bile duct and return to the intestine from the liver [52]. It is reported that SCFAs can be released into the liver through the portal vein and affect liver inflammation by regulating the expression of SCFA receptors in the liver [53]. Moreover, the other metabolites of the gut microbiota, including lipopolysaccharides, ethanol, ammonia, and acetaldehyde, can also promote the development of chronic hepatitis [54]. Disruption of the gut barrier allows for endotoxins, also known as lipopolysaccharides, to enter the bloodstream more easily. In the present study, a high dose of BPA exposure significantly decreased acetate, propionate, isobutyrate, valerate, and isovalerate content in feces, as well as the relative abundance of Bacteroidetes. The main fermentation products of Bacteroides are acetic acid, butyric acid, and succinic acid. Propionic acid and butyric acid can reduce the synthesis of cholesterol and triglyceride in the body [55]. Butyrate, which is the primary energy source for colonic epithelial cells, can reduce gut mucosal permeability and promote intestinal barrier recovery. Decreased production of butyric acid by gut flora is usually accompanied by an increase in opportunistic pathogens [56]. Thus, the reduced relative abundance of *Bacteroides* in this study reflected a disturbance of the gut microbiota. Furthermore, consistent with previous research results [57,58], exposure to BPA increased the abundance of gut *Proteobacteria* in rats. The change of *Proteobacteria* may further alter the structure and composition of tight junctions in the gut endothelium, leading to increased intestinal permeability and enhanced absorption of lipopolysaccharides into circulation [59]. *Prevotella* is symbiotic in the human intestines that degrade plant polysaccharides and synthesize vitamin B1 to reduce the incidence of autism [60]. As a beneficial bacterium, *Ruminococcaceae* can increase energy production from food in the gut, reduce the energy available for absorption, and is utilized as an energy source in the liver. The present study showed that at the genus level, the relative abundance of *Prevotella_9* and *Ruminococcaceae_UCG-014* were both significantly decreased in BPA-M and H groups. This means the liver injury induced by BPA may interlock with the disturbance of gut microbiota.

## 4. Materials and Methods

### 4.1. Animal Protocol

Thirty-two male Sprague Dawley (SD) rats, weighing approximately 100–120 g, were provided by Beijing HFK Bioscience Co., Ltd. (Certificate Number SCXK (jing) 2019-0008, Beijing, China). The animal experiment was approved by the Animal Care and Protection Committee of Jinan University, Guangzhou, China (Approval No. IACUC-20201027-12, Laboratory Animal Ethics Committee of Jinan University). The rats used in this study were cared for according to the National Institutes of Health Guide for the Care and Use of Laboratory Animals. Animals were housed at temperature (22–25 °C) and humidity (40–60%) with a 12/12 h light-dark cycle and had access to chow and water ad libitum. After 10 days of environmental acclimation, rats were randomly divided into four groups (*n* = 8 rats per group): the Control group (corn oil by oral gavage) and the low, medium, and high-dosage BPA-treated groups (30, 90, and 270 mg/kg bw BPA dissolved in corn oil, respectively). The no observed adverse effect level (NOAEL) and the lowest observable adverse effect level (LOAEL) of BPA were 5 mg/kg bw and 50 mg/kg bw, respectively [61,62]. For the treatment in this study, the “low dose” of 30 mg/kg bw was ≤50 mg/kg bw, and the “medium and high dose” of BPA was >50 mg/kg bw referring to previous literature [63]. BPA (analytical purity ≥ 99%, CAS: 80-05-7, Cat# 239658) was purchased from Sigma-Aldrich (St. Louis, MO, USA). The experiment lasted for 30 days. At the end of the experiment, the rats were anesthetized with sodium pentobarbital and blood was collected from the abdominal aorta for further analysis. Liver tissues were immediately removed and weighed. Parts of the liver were put into 4% paraformaldehyde solution for histopathological and immunological analysis, and the rest of the livers were stored at −80 °C until further investigation.

### 4.2. Chemicals

The primary antibodies Bax (Cat# AF1020), Bcl-2 (Cat# AF6139), TFAM (Cat# DF3232), and TNF-α (Cat# AF7014) were acquired from Affinity Biosciences (Cincinnati, OH, USA). Antibody for GAPDH (Cat# 2118S) was obtained from Cell Signaling Technology, Inc (Danvers, MA, USA). Antibodies for SIRT1 (Cat# 60303-1-Ig), PGC-1α (Cat# 66369-1-Ig), Nrf2 (Cat# 16396-1-AP), PARP1 (Cat# 13371-1-AP), and Caspase3 (Cat# 19677-1-AP) were purchased from Proteintech Group, Inc (Wuhan, China). Goat anti-rabbit IgG-HRP (Cat# 98164S) and anti-mouse IgG-HRP (Cat# 91196S) were purchased from Cell Signaling Technology, Inc (Danvers, MA, USA) and Proteintech Group, Inc (Wuhan, China), respectively.

### 4.3. Evaluation of Hepatic Oxidative Stress

To determine oxidative stress in liver tissue, 10% (*w*/*v*) homogenate was prepared in PBS buffer (pH 7.4) and centrifuged at 1520× *g* for 20 min at 4 °C. The activities of superoxide dismutase (SOD) (Cat# A001-3-2), glutathione (GSH) (Cat# A006-1-1), catalase (CAT) (Cat# A007-1-1), and malondialdehyde (MDA) (Cat# A003-1-2) were detected by commercial kits from Nanjing Jiancheng Bioengineering Institute. The protein concentration of liver samples was determined by bicinchoninic acid (BCA) (Cat# P1513-1) protein assay kit (Applygen Technologies Inc., Beijing, China).

### 4.4. Detection of Serum Biochemical Parameters

Blood samples were collected and placed at room temperature for 30 min. Then the serum was separated using a centrifuge at 1500× *g* for 15 min. Serum total cholesterol (TC) (Cat# A111-1-1), triacylglycerols (TG) (Cat# A110-1-1), low-density lipoprotein cholesterol (LDL-C) (Cat# A113-1-1), and high-density lipoprotein cholesterol (HDL-C) (Cat# A112-1-1) were tested by an automatic biochemical analyzer (HEMAVET 950FS, Drew Scientific, USA). The serum alanine and aspartate aminotransferase (ALT and AST) (Cat# C009-3-1, and C010-3-1) activity were measured by assay kits from Nanjing Jiancheng Bioengineering Institute (Nanjing, Jiangsu, China) following the provided protocols.

### 4.5. Histopathology and Immunohistochemistry of Liver

Liver tissues were fixed in 4% paraformaldehyde overnight. Then, the tissues were dehydrated in a gradient of ethyl alcohol solutions (75%, 85%, 90%, 95%, and 100%), after which the tissues were cleared by xylene. After impregnation with paraffin wax, the paraffin blocks were made. Next, the embedded tissues were cut into 4 μm thick sections and stained with hematoxylin-eosin (H&E) (Cat# GP1031).

Paraffin slices were immunohistochemically stained with SIRT1. In brief, the tissue sections were routinely dewaxed, dehydrated by ethanol gradient, and antigenic repair was performed with microwaved sodium citrate antigen repair solution. After blocking the nonspecific sites with 3% BSA, the sections were incubated with SIRT1 primary antibody (diluted 1:250) overnight at 4 °C. Subsequently, sections were incubated with the secondary antibody (Biotin-conjugated Affinipure Goat Anti-Mouse IgG (H+L), dilution 1:200, Cat# SA00004-1, Proteintech) for 50 min, followed by DAB staining and counterstaining with hematoxylin for 3 min. The sections were examined by a microscope (NIKON ECLIPSE E100, NIKON, Tokyo, Japan) equipped with an imaging system (NIKON DS-U3, NIKON, Tokyo, Japan) and the cumulative optical density was measured.

### 4.6. TUNEL Analysis

Terminal deoxynucleotidyl transferase-mediated dUTP nick end labeling (TUNEL) staining was performed. The paraffin-embedded liver sections were dewaxed and rehydrated, followed by treatment with proteinase K for 25 min, and washed with PBS. The sections were allowed to react with the labeling buffer, mixed with TDT and dUTP for 2 h at 37 °C, and washed with PBS. Apoptotic cells were stained green, while all nuclei were stained blue. TUNEL-positive cells were quantified under a microscope.

### 4.7. Real-Time PCR Analysis

Total RNA from the liver was extracted using Trizol reagent (Cat# R0016, Beyotime Institute of Biotechnology, Shanghai, China) following the manufacturer’s protocol and reverse transcribed into cDNA using a cDNA synthesis kit (Cat# AG11728, Accurate Biotechnology Co., Ltd., Hunan, China). The gene expressions of SIRT1, PGC-1α, Nrf1, Nrf2, TNF-α, and IL-1β were detected using an ABI QuantStudio™ 6 system (Applied Biosystems; Thermo Fisher Scientific, Inc. (Waltham, MA, USA)) with the SYBR Green PCR kit (Cat# AG11718, Accurate Biotechnology Co., Ltd., Changsha, China). The primers were synthesized by Shanghai Sangon Biological Engineering Co., Ltd. (Shanghai, China) Relative quantification was calculated using the 2^−∆∆Ct^ method. Primer sequences are shown in Table 1.

### 4.8. Western Blotting Analysis

Liver tissues (100 mg) were homogenized in 1 mL RIPA (Cat# P0013B, Beyotime, Shanghai, China) buffer containing the protease inhibitor (Cat# P1010, Beyotime, Shanghai, China) and phenylmethanesulfonyl fluoride (PMSF) (Cat# ST506, Beyotime, Shanghai, China) and centrifuged for supernatants at 14,000× *g* for 10 min at 4 °C. Subsequently, the protein concentration was measured by using BCA Protein Assay Kit (Cat# P0012, Beyotime, Shanghai, China). Protein samples were separated by 10% SDS-polyacrylamide gel and then transferred to the PVDF membrane (Cat# ISEQ00010, Millipore, MA, USA). The blots were blocked with 5% skimmed milk at room temperature for 1.5 h, then membranes were incubated with primary antibodies for Bax (1:1000), Bcl-2 (1:1000), GAPDH (1:1000), TFAM (1:1000), TNF-α (1:1000), PARP1 (1:1000), SIRT1 (1:1000), PGC-1α (1:2000), Nrf2 (1:1000), and Caspase3 (1:1000) at 4 °C, and subsequently incubated with goat anti-rabbit IgG-HRP (1:2000) and anti-mouse IgG-HRP (1:5000) secondary antibodies. Finally, protein bands were visualized using ECL Reagent (Cat# JP001B250, Clinx Science Instruments, Shanghai, China) and the gray value of the bands was calculated automatically by the Clinx ChemiScope working system (Model No: 5300, Clinx Science Instruments, China).

### 4.9. Gut Microbiota Analysis by High-Throughput 16S rRNA Gene Sequencing

Total DNA of feces was extracted by using the MagPure Universal RNA LQ Kit (Cat# R6623, Magen Biotechnology Co., Ltd., Guangzhou, China) according to the manufacturer’s directions. The DNA concentration and purity were controlled using NanoDrop 2000 spectrophotometry (Thermo Fisher Scientific Inc, Waltham, MA, USA) and agarose gel electrophoresis. The V3-V4 variable region of the 16S rRNA genes was amplified using the universal primers 338F (5′-ACTCCTACGGGAGGCAGCA-3′) and 806R (5′-GGACTACHVGGGTWTCTAAT-3′). Amplicons were purified using the Agencourt AMPure XP system (Cat# A63881, Beckman Coulter, Inc. (Brea, CA, USA) and pooled in an equimolar amount. Then amplicons were subjected to pyrosequencing using the Illumina NovaSeq 6000 sequencing platform with a paired-end read of 2 × 250 cycles according to standard protocols13 (Illumina Inc., San Diego, CA, USA; OE Biotech Company; Shanghai, China). For bioinformatic analysis, raw sequencing data were in FASTQ format. Paired-end reads were then preprocessed and cut off ambiguous bases (N). It also cut off low-quality sequences with an average quality score below 20 using the sliding window trimming approach. After trimming, paired-end reads were assembled using FLASH software. Further processing of paired-end reads including quality filtering and removal of mismatched barcodes, and sequences were completed using QIIME (version 1.8.0, Gregory Caporaso, Flagstaff, Arizona). Clean reads were subjected to primer sequence removal and clustering to generate operational taxonomic units (OTUs) using Vsearch software with a 97% similarity cutoff. All representative reads were annotated and blasted against Silva database Version 132.

### 4.10. Analysis of Fecal Short Chain Fatty Acids (SCFAs)

Fecal SCFAs were analyzed by using gas chromatography (Shimadzu G2010Plus, Kyoto, Japan) equipped with a DB-FFAP chromatographic capillary column (30 m × 0.530 mm × 1.00 μm; Agilent, Santa Clara, CA, USA). The pre-treatment of samples was based on a method described before with some modifications [64]. In brief, fresh feces (100–150 mg) were dissolved in 0.8 mL 0.2 M HCl (0.02 mg/mL 2-ethylbutyric acid as internal standard) and 0.2 mL 0.15 M oxalate. The mixture was vortexed for 1 min and centrifuged at 12,000× *g* and 4 °C for 15 min. The supernatant was filtrated using a 0.22 μm filter for further analysis. N2 was supplied as carrier gas at a flow rate of 10 mL/min. The flow rates of air, H2, and N2 as make up gas were 260, 30, and 30 mL/min, respectively. The oven temperature was increased from 100 °C to 160 °C at 5 °C/min and then held at this temperature for 4 min. The SCFAs contents, including acetate, propionate, isobutyrate, butyrate, valerate, and caproate were quantified using standard curves.

### 4.11. Statistical Analysis

Data were expressed as mean ± standard error of the mean (SEM). SPSS 23.0 (SPSS Inc., Chicago, IL, USA) and GraphPad Prism 8.0 software (San Diego, CA, USA) was used for statistical analysis and graphical presentation. A significant difference was analyzed by one-way analysis of variance (ANOVA). In post hoc analysis, if variances were homogeneous, the Bonferroni test was applied. If not, the Games–Howell test was used for analysis. Values were considered to be significantly different when *p* < 0.05.

## 5. Conclusions

**Figure 8:**
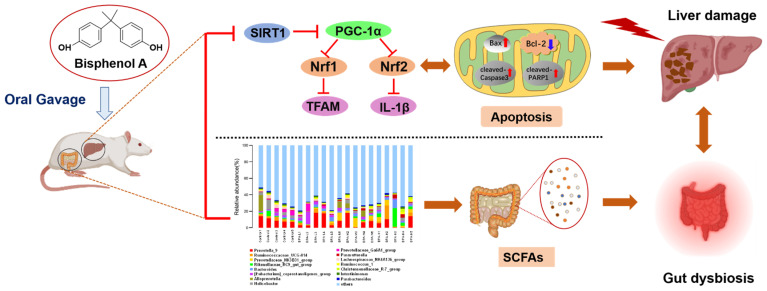
Schematic diagram of the mechanism of BPA-induced liver injury and gut microbiota dysbiosis in rat.

In conclusion, the present study indicated that BPA-induced liver toxicity by inducing oxidative stress, promoting mitochondrial apoptosis, and inhibiting the SIRT1/PGC-1α signaling pathway. In addition, BPA exposure also led to the disturbance of intestinal flora and the reduction of SCFAs levels, which is associated with hepatoxicity (Figure 8). These findings offer novel insights into the emerging research area to evaluate the impact of BPA on the liver and intestinal flora. The in-depth mechanism between microbiome and liver injury induced by BPA needs to be further explored through added antibiotics.

## Figures and Tables

**Table 1:** Primer sequences for quantitative RT-PCR analyses.

Gene Name	Primers Sequences (5′-3′)	Product Size (bp)	Annealing Temperature	Accession Number
*SIRT1*	Forward: GCTCGCCTTGCTGTGGACTTCC Reverse: GTGACACAGAGATGGCTGGAACTG	141	60 °C	NM_001372090.1
*PGC-1α*	Forward: CATTCAGGAGCTGGATGGCT Reverse: AGATCTGGGCAAAGAGGCTG	106	60 °C	NM_031347.1
*Nrf1*	Forward: GGCGCAGCACCTTTGGAGAATGTG Reverse: CATCGATGGTGAGAGGGGGCAGTTC	133	60 °C	NM_001100708.1
*Nrf2*	Forward: GAGACGGCCATGACTGA Reverse: GTGAGGGGATCGATGAGTAA	196	60 °C	NM_031789.2
*TNF-α*	Forward: CCACGCTCTTCTGTCTACTG Reverse: GCTACGGGCTTGTCACTC	145	60 °C	NM_012675.3
*IL-1β*	Forward: TCTGTGACTCGTGGGATGAT Reverse: CTTCTTTGGGTATTGTTTGG	181	60 °C	NM_031512.2
*β-actin*	Reverse: CCCAGGCATTGCTGACAGGATG Forward: TGCTGGAAGGTGGACAGTGAGG	144	60 °C	NM_031144.3

## Data Availability

All data presented in this study are available in the main body of the manuscript.

## Abbreviations

PGC-1α	Peroxisome proliferator-activated receptor-gamma-coactivator 1alpha
Nrf1	Nuclear respiratory factor
Nrf2	Nuclear factor E2-related factor 2
TFAM	Mitochondrial transcription factor A
NOAEL	No observed adverse effect level
LOAEL	The lowest observable adverse effect level
ROS	Reactive oxygen species
SOD	Superoxide dismutase
GSH	Glutathione
CAT	Catalase
MDA	Malondialdehyde
TC	Total cholesterol
TG	Triacylglycerols
LDL-C	Low-density lipoprotein cholesterol
HDL-C	High-density lipoprotein cholesterol
ALT	Alanine aminotransferase
AST	Aspartate aminotransferase
TUNEL	Terminal deoxynucleotidyl transferase-mediated dUTP nick-end labeling
SCFAs	Short-chain fatty acids
GST	Glutathione S-transferase
GSH-Px	Glutathione peroxidase
PCA	Principal component analysis
ALP	Alkaline phosphatase
LDH	Lactate dehydrogenase
